# 17-DMAG对EGFR-TKI耐药的非小细胞肺癌细胞株A549和H1975作用的研究

**DOI:** 10.3779/j.issn.1009-3419.2014.11.02

**Published:** 2014-11-20

**Authors:** 雷 赵, 富民 曹

**Affiliations:** 050011 石家庄，河北医科大学第四医院胸三科 Department of Thoracic Surgery, the Fourth Hospital of Hebei Medical University, Shijiazhuang 050011, China

**Keywords:** 肺肿瘤, 热休克蛋白90抑制剂, 表皮生长因子受体酪氨酸激酶抑制剂, 增殖, 凋亡, Lung neoplasms, HSP90 inhibitors, EGFR-TKI, Proliferation, Apoptosis

## Abstract

**背景与目的:**

表皮生长因子受体酪氨酸激酶抑制剂（epidermal growth factor receptor-tyrosine kinase inhibitor, sEGFR-TKI）在非小细胞肺癌（non-small cell lung cancer, NSCLC）患者的临床治疗中产生的原发性及获得性耐药限制了其临床应用，需要探索新的策略或方法来克服这个问题。最近有文献报道认为热休克蛋白90（heat shock protein 90, HSP90）抑制剂能从多种途径和环节发挥抗肿瘤作用，这为解决NSCLC对EGFR-TKI的耐药提供了新的思路。本研究通过观察HSP90抑制剂17-DMAG对EGFR-TKI分别原发性及获得性耐药的NSCLC细胞株A549和H1975的作用，旨在探讨它对细胞增殖、凋亡与EGFR蛋白表达的影响及其可能的机制。

**方法:**

以不同浓度的17-DMAG分别作用于A549和H1975细胞株24 h、48 h、72 h，应用四甲基偶氮唑蓝（MTT）比色法检测细胞增殖；作用48 h后，应用流式细胞术PI单染法检测细胞凋亡，并应用Western blot检测细胞HSP90及EGFR蛋白表达水平。

**结果:**

17-DMAG在不同药物浓度和作用时间对A549和H1975细胞的增殖抑制率差异均有统计学意义（*P*＜0.01），且呈时间和剂量依赖性；两种细胞不同药物浓度组和空白对照组之间的凋亡率差异均有统计学意义（*P*＜0.01），且呈剂量依赖性；17-DMAG作用48 h后，A549细胞的EGFR/GADPH和HSP90/GADPH及H1975细胞的HSP90/GADPH在不同药物浓度组和空白对照组之间的灰度比值差异均无统计学意义（*P*＞0.05），而H1975细胞的EGFR/GADPH在不同药物浓度组和空白对照组之间的灰度比值差异均有统计学意义（*P*＜0.01）。

**结论:**

17-DMAG对NSCLC细胞株A549和H1975均具有抑制增殖及促进凋亡作用，且它能降低突变型EGFR的蛋白表达水平，而对野生型EGFR的蛋白表达无明显影响。本研究为EGFR-TKI耐药的非小细胞肺癌提供了新的治疗策略。

## Abstract (en)

**Background and objective:**

In the clinical treatment of patients with non-small cell lung cancer (NSCLC), the primary and acquired resistance of epidermal growth factor receptor-tyrosine kinase inhibitor (EGFR-TKI) limits its clinical application, this need to explore new strategy or method to overcome this problem. Recently, some literatures have indicated that the antitumor role of heat shock protein 90 (HSP90) inhibitors by a variety of pathways may provide new strategy for resolving this problem. In this study, we examined the effect of 17-DMAG on NSCLC cell lines A549 and H1975 which were primary and acquired resistant to EGFR-TKI respectively, the purpose was to explore its influence on cell proliferation, apoptosis and the expression of EGFR *in vitro* as well as possible mechanism.

**Methods:**

After A549 and H1975 cell lines were treated with different concentrations of 17-DMAG respectively, the inhibitory rate of cell proliferation was measured by MTT assay in 24 h, 48 h and 72 h. We investigated the effect of 17-DMAG on the cell apoptosis with flow cytometry and the expression of HSP90 and EGFR with Western blot after treated with 17-DMAG for 48 h.

**Results:**

After treated with 17-DMAG, the inhibitory rate of different concentrations and time groups was significant (*P* < 0.01), and the effect was in time- and dose-dependent manner; the apoptosis rate of both two cell lines in all treated groups were significantly higher than control group (*P* < 0.01), and the effect was in dose-dependent manner. By Western blot analysis, there was no significant difference between all treated groups and control group for the expression of both HSP90 and EGFR protein in A549 cell line and HSP90 protein in H1975 cell line after exposed to 17-DMAG for 48 h (*P* > 0.05), while the difference was significant for the expression of EGFR protein in H1975 cell line (*P* < 0.01).

**Conclusion:**

17-DMAG inhibited the proliferation of NSCLC cell lines A549 and H1975 and also induced apoptosis of both cell lines. It down-regulated the expression of mutant EGFR protein while this phenomenon was not observed in EGFR-wild type cell line. This suggested that the mechanism maybe different between A549 and H1975 cell lines with different genetic backgroud. Our study provided new strategy for treatment with NSCLC being resistant to EGFR-TKI.

近年来以吉非替尼（gefitinib, iressa）和厄洛替尼（erlotinib, tarceva）为代表的表皮生长因子受体酪氨酸激酶抑制剂（epidermal growth factor receptor-tyrosine kinase inhibitor, EGFR-TKI）作为分子靶向药物已正式应用于非小细胞肺癌（non-small cell lung cancer, NSCLC）患者的临床治疗，并在一定程度上延长了部分患者的生存期^[1]^。然而，约60%的NSCLC患者对此类药物并不敏感，并且几乎所有初始对其有效的患者最终都会出现疾病进展，即分别发生了原发性及获得性耐药^[2]^。因此，探索EGFR-TKI耐药的机制及克服策略已成为肺癌治疗领域的研究热点。

最近国外有研究^[3]^认为，EGFR在肺癌中的作用与HSP90密切相关，抑制HSP90能从多种途径和环节发挥抗肿瘤作用，这为解决NSCLC对TKI的耐药提供了新的思路。但HSP90抑制剂对于EGFR-TKI原发性及获得性耐药的不同NSCLC是否均具有抗肿瘤作用，至今尚未形成统一结论^[4-6]^，而国内未发现相关报道。本研究将HSP90抑制剂17-DMAG作用于对EGFR-TKI原发性及获得性耐药具有代表性的NSCLC细胞株A549和H1975，观察它在体外对细胞增殖、凋亡及EGFR蛋白表达的影响，并对其机制进行初步探讨，从而为HSP90抑制剂在EGFR-TKI耐药的NSCLC中的临床应用提供实验依据。

## 1 材料与方法

### 1.1 主要材料与仪器

人NSCLC细胞株A549和H1975分别购自北京协和细胞资源中心及中国科学院上海生命科学研究院细胞资源中心；RPMI-1640细胞培养液购自美国GIBCO公司；17-DMAG购自美国Selleck公司；四甲基偶氮唑蓝（MTT）购自美国Amresco公司；EGFR抗体购自美国Epitomics公司；HSP90抗体购自美国CST公司；GADPH抗体购自美国Invitrogen公司；全自动酶标仪由奥地利Termo公司生产；流式细胞仪由美国BD公司生产；红外荧光扫描成像系统由美国LI-COR公司生产。

### 1.2 细胞培养

A549和H1975细胞均使用含10%胎牛血清的RPMI-1640培养液（内含青霉素100 U/mL、链霉素100 μg/mL）置于37 ℃、5%CO_2_、饱和湿度培养箱中进行培养，根据细胞生长情况每2-3天用0.25%胰酶常规消化传代，取对数生长期细胞进行实验。

### 1.3 MTT法检测17-DMAG对细胞的增殖抑制作用

取对数生长期细胞，调整细胞密度为6×10^4^个/mL，以每孔90 μL的体积接种于96孔细胞培养板，实验设正常细胞对照组和不同药物浓度作用实验组，每组均设6个复孔。次日细胞贴壁后，对照组每孔加入10 μL不含胎牛血清的RPMI-1640培养液，实验组每孔加入17-DMAG溶液10 μL，使药物终浓度为0.003 μmol/L、0.03 μmol/L、0.3 μmol/L、3 μmol/L、30 μmol/L。培养24 h、48 h、72 h后，每孔加入5 mg/mL的MTT溶液10 μL，继续培养4 h后加入DMSO溶液150 μL，用全自动酶标仪测定各孔在波长570 nm处的吸光度（optical density, OD）值。按下式计算细胞增殖抑制率：抑制率=（1-实验组OD值/对照组OD值）×100%。

### 1.4 流式细胞术检测细胞凋亡

取对数生长期细胞，随机分为对照组和实验组，细胞贴壁后用PBS缓冲液冲洗，每组细胞加入含10%胎牛血清的RPMI-1640培养液4.5 mL，对照组加入不含胎牛血清的RPMI-1640培养液0.5 mL，各实验组加入17-DMAG溶液0.5 mL，使药物终浓度为0.003 μmol/L、0.03 μmol/L、0.3 μmol/L、3 μmol/L。培养48 h后PBS洗涤并用胰酶消化后离心去上清，每组加入70%乙醇溶液3 mL，4 ℃固定过夜。次日弃去乙醇，PBS离心洗涤两遍，加入碘化丙啶（PI）染液0.3 mL-0.5 mL重悬细胞后4 ℃避光染色30 min。每组样品至少收集10^6^个细胞，流式细胞仪上机检测细胞凋亡率。

### 1.5 Western blot检测细胞EGFR及HSP90蛋白表达水平

取对数生长期细胞，随机分为对照组和实验组，各实验组17-DMAG作用的终浓度为0.003 μmol/L、0.03 μmol/L、0.3 μmol/L、3 μmol/L、30 μmol/L。培养48 h后，提取对照组及各实验组蛋白，根据BCA法蛋白定量试剂盒说明测定蛋白浓度，将蛋白样品加入SDS-聚丙烯酰胺凝胶，每孔上样量为50 μg，经电泳、转膜、5%脱脂奶粉封闭后加入一抗（1:3, 000），4 ℃孵育过夜，加入荧光二抗（1:5, 000）37 ℃避光孵育1 h，应用红外荧光扫描成像系统对目的蛋白条带图像进行分析，以EGFR/GADPH和HSP90/GADPH的灰度比值表示A549和H1975细胞中两种蛋白的相对表达量。

### 1.6 统计学分析

全部独立实验均重复3次，结果以均数±标准差（Mean±SD）表示，应用SPSS 16.0软件对数据进行分析，Excel 2003作图。各组间的均数比较采用单因素方差分析，以*P*＜0.05为差异具有统计学意义。

## 2 结果

### 2.1 17-DMAG对A549和H1975细胞的增殖抑制作用

MTT法检测结果显示，以终浓度0.003 μmol/L、0.03 μmol/L、0.3 μmol/L、3 μmol/L、30 μmol/L的17-DAMG作用24 h、48 h、72 h后，17-DAMG在不同药物浓度和不同作用时间对A549和H1975细胞的增殖抑制率差异均有统计学意义（*P*＜0.01），且随着药物浓度的增加和作用时间的延长，抑制率逐渐增大，呈时间和剂量依赖性（表 1）。

**1:** 17-DAMG在不同浓度和时间对细胞株的增殖抑制作用 The inhibitory effect of 17-DMAG on proliferation of cell lines treated by different concentrations and time

Cell lines	17-DMAG (*μ*mol/L)	Inhibitory rate (%)			*P*
		24 h	48 h	72 h	
A549	0.003	10.51±1.65	21.89±1.57	29.15±1.19	＜0.001^**^
	0.03	17.60±1.43	28.76±3.06	38.64±2.31	＜0.001^**^
	0.3	32.32±2.72	38.45±1.74	60.23±1.26	＜0.001^**^
	3	42.58±3.31	57.38±1.21	78.45±1.34	＜0.001^**^
	30	54.49±0.95	73.73±2.42	88.90±0.89	＜0.001^**^
	*P*	＜0.001^*^	＜0.001^*^	＜0.001^*^	
H1975	0.003	8.75±2.43	16.43±2.63	28.81±3.06	＜0.001^**^
	0.03	13.50±1.54	28.22±2.19	39.68±1.05	＜0.001^**^
	0.3	22.71±3.54	40.30±5.23	56.26±2.50	＜0.001^**^
	3	45.21±3.84	59.82±2.05	75.33±3.08	＜0.001^**^
	30	70.51±2.41	81.17±5.38	90.61±2.51	0.002^**^
	*P*	＜0.001^*^	＜0.001^*^	＜0.001^*^	
^*^: *P* value in groups of different concentrations.^**^: *P* value in groups of different time.					

### 2.2 17-DMAG对A549和H1975细胞凋亡的影响

流式细胞术结果显示，以终浓度0.003 μmol/L、0.03 μmol/L、0.3 μmol/L、3 μmol/L的17-DAMG作用48 h后，17-DMAG对A549和H1975细胞的凋亡率均高于对照组，不同药物浓度组和对照组之间的凋亡率差异均有统计学意义（*P*＜0.01），且凋亡率随着药物浓度的增加而逐渐增大，呈剂量依赖性（图 1）。

**1:**
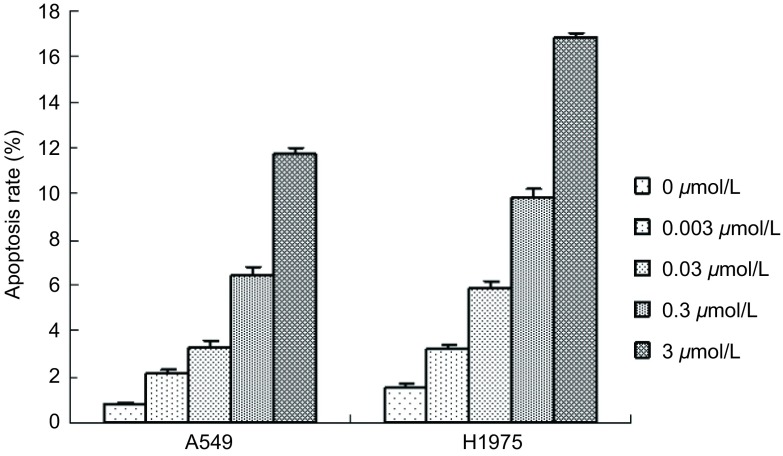
17-DMAG对细胞凋亡的影响 The effect of 17-DMAG on cell apoptosis

### 2.3 17-DMAG对A549和H1975细胞HSP90及EGFR蛋白表达的影响

以终浓度0.003 μmol/L、0.03 μmol/L、0.3 μmol/L、3 μmol/L、30 μmol/L的17-DAMG作用48 h后，A549细胞不同药物浓度组和对照组之间的HSP90/GADPH及EGFR/GADPH灰度比值差异均无统计学意义（*P*＞0.05）；H1975细胞不同药物浓度组和对照组之间的HSP90/GADPH灰度比值差异均无统计学意义（*P*＞0.05），而EGFR/GADPH灰度比值差异均有统计学意义（*P*＜0.01）。以上结果表明，17-DMAG对A549和H1975细胞的HSP90及A549细胞的EGFR蛋白表达均无明显影响，但它能降低H1975细胞的EGFR蛋白表达水平，且随着药物浓度的增加逐渐降低（图 2）。

**2:**
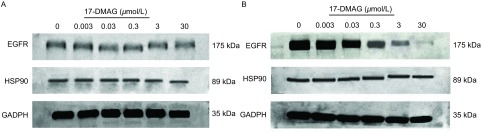
17-DMAG对细胞株EGFR和HS90蛋白表达的影响。A: A549细胞株；B: H1975细胞株。 The effect of 17-DMAG on protein expression of EGFR and HSP90 of cell lines. A: A549 cell line; B: H1975 cell line.

## 3 讨论

NSCLC对EGFR-TKI耐药的作用机制涉及多种基因水平改变和信号转导通路异常激活。HSP90是广泛存在于生物体内的多功能分子伴侣，它的“客户蛋白”大多是肿瘤细胞增殖、分化、侵袭、转移及血管生成等重要过程所依赖的蛋白激酶、转录因子或癌基因的产物，包括突变的EGFR、血管内皮生长因子受体（vascular endothelial cell growth factor receptor, VEGFR）、人表皮生长因子受体2（human epidermal growth factor 2, HER2）、survivin、缺氧诱导因子-1α（hypoxia inducible factor-1, HIF-1α）及细胞周期调节因子（CDK4、CDK6）等^[7]^。抑制HSP90可导致这些“客户蛋白”通过泛素-蛋白酶体途径发生降解，从多种途径和环节产生抗肿瘤作用而实现多靶点攻击^[3]^。

本研究中，A549细胞株为野生型EGFR，同时含有*K*-*ras*基因突变。约10%-30%的NSCLC患者存在*K*-*ras*基因突变，它是导致EGFR-TKI原发性耐药的重要机制。Pao等^[8]^用TKI治疗60例肺腺癌患者时发现38例不敏感的患者有9例存在*K*-*ras*基因突变，Massarelli等^[9]^认为*K*-*ras*突变和*EGFR*突变相互排斥，是评价NSCLC患者能否接受TKI治疗的阴性预测因子。H1975细胞株的EGFR含有L858R和T790M双突变。L858R为EGFR第21外显子858位点亮氨酸置换为精氨酸的点突变，是TKI治疗的敏感突变。T790M是发生在EGFR第20外显子790位密码子苏氨酸置换为甲硫氨酸的错义突变，NSCLC对EGFR-TKI的获得性耐药50%以上是由*T790M*突变造成的^[10-12]^，它是国际公认的重要获得性耐药机制。

我们应用体外实验的方法，将HSP90抑制剂17-DMAG作用于两种遗传背景不同的NSCLC细胞株A549和H1975，结果表明它对两种细胞均产生了明显的增殖抑制作用，与Shimamura等^[5]^的研究结果相似，而Kobayashi等^[6]^则认为17-DMAG不能抑制A549细胞增殖，可能与本实验药物作用剂量梯度不同有关。A549细胞的*K*-*ras*突变直接持续激活Ras-Raf-MEK-MAPK信号通路，并失去对上游EGFR的依赖性，导致TKI耐药的产生，而H1975细胞依赖*EGFR*突变后异常激活的多条下游信号转导通路维持生存和增殖，HSP90抑制剂能同时阻断这些信号通路从而抑制细胞增殖。本研究显示17-DMAG能促进A549和H1975细胞凋亡，作用48 h后的平均凋亡率分别为11.73%和16.88%，造成总体凋亡率较低的原因可能与细胞状态、处理过程及检测方法等因素有关。研究表明，细胞周期蛋白依赖性蛋白激酶家族的CDK4和CDK6是HSP90的“客户蛋白”，抑制HSP90可以降低它们的表达水平，使细胞出现生长周期阻滞并发生凋亡^[13]^。Survivin是目前已知最强的凋亡抑制因子，在多种恶性肿瘤中呈高表达状态，抑制HSP90能使它发生降解，从而促进肿瘤细胞凋亡。这些都可能是17-DMAG作用后细胞发生凋亡的原因。此外，我们发现17-DMAG对两种细胞HSP90的表达均无明显影响，再次证实它只是发挥分子伴侣的作用，其本身在药物作用之后并不会发生降解。相关研究^[14]^认为，HSP90抑制剂可下调A549细胞先前存在或重新激活的HER2及PI3K/Akt和STAT3/STAT5信号级联反应中的关键调节因子的表达，而与野生型EGFR的存在无关，这可能是EGFR在17-DMAG作用后未发生明显降解的原因。而H1975细胞中突变的EGFR需要HSP90来维持它的构象成熟和状态稳定以保证正常功能的发挥，所以对于HSP90抑制剂引起的降解效应更为敏感。相关的具体作用机制尚有待于进一步证实。

近年来，HSP90抑制剂作为多靶点的抗肿瘤药物因其独特的优越性已在各种恶性肿瘤治疗方面进行了不同时期的临床试验。随着肺癌的发病机制不断阐明，根据患者的自身情况和基因状态设计合理的用药方案从而实现真正的个体化治疗是未来肺癌临床研究的前沿和热点，HSP90抑制剂也将越来越受到人们的关注并发挥重要作用。
